# Double Disadvantage in a Nordic Welfare State: A Demographic Analysis of the Single-Parent Employment Gap in Finland, 1987–2018

**DOI:** 10.1007/s10680-023-09651-w

**Published:** 2023-02-21

**Authors:** Juho Härkönen, Marika Jalovaara, Eevi Lappalainen, Anneli Miettinen

**Affiliations:** 1 European University Institute, Florence, Italy; 2 Department of Social Research, University of Turku, Turku, Finland; 3 Social Insurance Institution, Helsinki, Finland; 4 Stockholm University, Stockholm, Sweden

**Keywords:** Single mothers, Single fathers, Single parents, Employment, Inequality, Education, Finland

## Abstract

This study demonstrates how an evolving negative educational gradient of single parenthood can interact with changing labour market conditions to shape labour market inequalities between partnered and single parents. We analysed trends in employment rates among Finnish partnered and single mothers and fathers from 1987 to 2018. In the late 1980s’ Finland, single mothers’ employment was internationally high and on par with that of partnered mothers, and single fathers’ employment rate was just below that of partnered fathers. The gaps between single and partnered parents emerged and increased during the 1990s recession, and after the 2008 economic crisis, it widened further. In 2018, the employment rates of single parents were 11–12 percentage points lower than those of partnered parents. We ask how much of this single-parent employment gap could be explained by compositional factors, and the widening educational gradient of single parenthood in particular. We use Chevan and Sutherland’s decomposition technique on register data, which allows us to decompose the single-parent employment gap into the composition and rate effects by each category of the background variables. The findings point to an increasing double disadvantage of single parents: the gradually evolving disadvantage in educational backgrounds together with large differences in employment rates between single and partnered parents with low education explain large parts of the widening employment gap. Sociodemographic changes in interaction with changes in the labour market can produce inequalities by family structure in a Nordic society known for its extensive support for combining childcare and employment for all parents.

**Supplementary Information:**

The online version contains supplementary material available at 10.1007/s10680-023-09651-w.

## Introduction

**Fig. 1:**
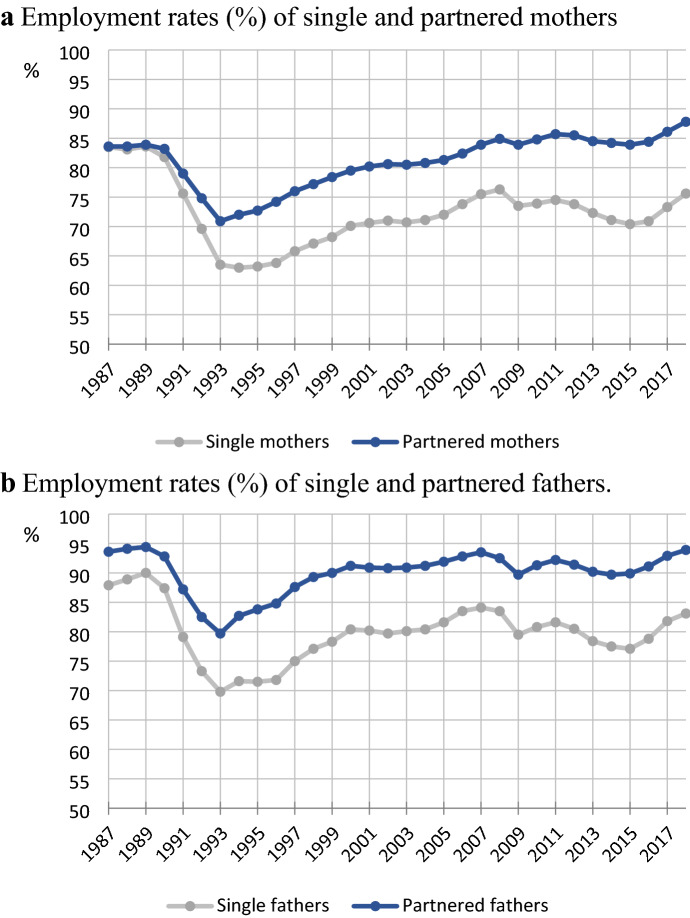
Employment rates (%) of **a** single and partnered mothers and **b** single and partnered fathers, 1987–2018

Finnish single parents’ employment rates were high and similar to those of partnered parents up until the early 1990s economic recession, which corresponded to the view of Finland as a Nordic welfare regime that supports the employment of all parents regardless of gender or family status (e.g., Esping-Andersen, 1999; Nieuwenhuis & Maldonado, 2018). As shown in Fig. 1, the situation changed following the 1990s economic recession, which led to a major decline in single parents’ employment in particular. The employment gap between partnered and single parents has remained large since despite an improved labour market. This development has gone largely undocumented in research into single parents’ employment (e.g. Hakovirta, 2006).

This objective of this paper is to contribute to understanding the social demographic sources of the change in the single-parent employment gap. In particular, we focus on the changing educational gradient of single parenthood, a central theme of the past two decades of social demographic research (e.g., McLanahan, 2004; McLanahan & Jacobsen, 2015). This change has been characterised by an increase in the prevalence of single parenthood among the low educated (Härkönen, 2017), which has weakened single parents’ educational profile relative to partnered parents. The impact of this increasing prevalence can be amplified if the employment penalty associated with single parenthood is higher among the less educated (cf. Brady et al., 2017; Zagel, et al., 2021). The changing educational gradient of single parenthood may thus lead to a double disadvantage, where single parents’ weaker educational profile is coupled with a educationally stratified single-parent employment gap.

Our paper contributes to understanding the changes in single parents’ employment over the past decades, and to research on single parents’ well-being at a time of social demographic change more generally (McLanahan, 2004; McLanahan & Percheski, 2008). It provides one of the few empirical assessments of how family bifurcation shapes inequality (cf. Bernardi & Boertien, 2017; Härkönen, 2018; Zagel, et al., 2021). We analyse both single mothers and single fathers. The literature on single parenthood has largely overlooked the increase in single-father families (Nieuwenhuis, 2020), which face similar economic hardships as single mothers (Chzhen & Bradshaw, 2012; Maldonado & Nieuwenhuis, 2015; Semega et al., 2017). We use Chevan and Sutherland’s (2009) decomposition technique on high-quality Finnish population registers, which allows us to consider the contributions of employment gaps at each level of education.

## Background

### The Social Demography of Single Parenthood

Single parenthood is still largely single motherhood, single-mother households constituting 85% of all Finnish single-parent households. The share of single-mother families of all families with children under 18 years of age increased from 12% in 1990 to 20% in 2019 (Statistics Finland, 2021). Single-father families remain a minority, comprising less than 2% of all families with children in 1990 and 3.4% in 2019 (Statistics Finland, 2021).

The large majority of Finnish single motherhood results from separations rather than out-of-union childbearing (Heuveline et al., 2003; Jalovaara & Andersson, 2018). Consequently, the average age of Finnish single mothers is similar to that of partnered mothers, but the former are less likely to have very young children. This is in contrast to the USA and the UK, for instance, where single mothers’ younger age is one reason for their low employment (Ahn, 2012; Wu & Eamon, 2011). Becoming a single father without a previous union is rare (Capková & Jalovaara, 2020). In our data, single fathers tend to be slightly older than partnered fathers and less likely to have very young children.

Because divorce and separation (Jalovaara, 2013) as well as births outside coresidential partnership (Jalovaara & Andersson, 2018) are more common among the less than the highly educated, single parenthood is more common among lower-educated mothers in Finland as well as many other countries (Härkönen, 2017; McLanahan, 2004). This gap emerged during the past decades. It was next to absent in the 1980s, but began to widen thereafter as single motherhood increased among mothers with medium, and especially, low levels of education, but remained stable among highly educated mothers (Härkönen, 2017). Although knowledge on the relationship between education and single fatherhood is less extensive, findings of negative (Brown, 2000; Capková & Jalovaara, 2020; Eggebeen et al., 1996; Galarneau, 2005) and growing (Galarneau, 2005) socioeconomic gaps and the negative educational gradients of union dissolution among men (Jalovaara & Kulu, 2018) suggest similar educational patterns as for single mothers.

### Single Parents and Employment

The standard economic model of labour supply holds that parenthood affects mothers’ and fathers’ employment differently. Motherhood is expected to decrease labour supply, because having children increases the value of women’s time outside paid work (Bargain et al., 2014; Cahuc et al., 2014). Fathers, on the other hand, take less time off from paid work and may instead increase their work effort to meet the increasing financial obligations (e.g., Killewald & Gough, 2013; Knoester & Eggebeen, 2006; Petersen et al., 2014). In addition to differential time investments, employers may discriminate against mothers (Correll et al., 2007) but favour fathers (Bygren & Gähler, 2012).

Research on single parents’ employment has concentrated on mothers. In general, single and partnered mothers’ employment is affected by similar factors. Both are more likely to be employed if they are highly educated and have higher potential earnings, fewer and older children, good access to high-quality child care, and lower incomes from social benefits or other non-work sources (e.g. Ahn, 2012; González, 2004; Gornick, 2004; Misra et al., 2012; OECD, 2011; Rafferty & Wiggan, 2011; Steiber & Haas, 2012; Wu & Eamon, 2011). Differences in these factors partly explain the differences between single and partnered mothers’ employment rates (González, 2004; OECD, 2011; Wu & Eamon, 2011).

As sole family earners, single mothers have higher work incentives than partnered mothers (González, 2004). However, single mothers’ labour supply can be more responsive to wage rates and non-work income (Bargain et al., 2014), not least because their childcare responsibilities make them more dependent on access to affordable childcare (Connelly & Kimmel, 2003; Misra et al., 2012). Childcare responsibilities can also reduce single mothers’ opportunities and willingness to accept job offers with non-typical working hours (Esser & Olsen, 2017; Moilanen et al., 2019) or reduce employers’ willingness to hire them. Furthermore, because single mothers more often receive means-tested social benefits such as unemployment and housing benefits and social assistance, single mothers can face stronger employment disincentives (e.g. Thévenon, 2011). Even at equal social benefit levels, single mothers’ labour supply can be more affected more by non-work income if their labour supply is more elastic (Bargain et al., 2014; Mastrogiacomo et al., 2013).

The above discussion suggests that the single-mother employment gap is likely to be larger in the lower educational segments. First, less educated mothers receive lower wages than highly educated mothers. They are thus more likely to be faced with employment disincentives due to a constellation of different (means-tested) social benefits, and these disincentives can be larger for single mothers than for partnered mothers because of differences in benefit packages as well possibly higher labour supply elasticity among the former than the latter (Bargain et al., 2014; Mastrogiacomo et al., 2013; Thévenon, 2011). Second, mothers with lower levels of education more often face less family-friendly working hours and other conditions (Presser & Ward, 2011). The effect of working conditions on employment is likely stronger among single mothers, who tend to face more constraints in arranging childcare (cf. Kjeldstad & Rønsen, 2004).

Much of the research on single fathers has focused on their caregiving as well as their and their children’s well-being (Coles, 2015), and this scholarship provides cues to theorise single fathers’ employment. The microstructural perspective argues that family status trumps gender to produce similar effects of single parenthood for women and men alike (Hook & Chalasani, 2008). Since single parents have fewer opportunities to divide paid and unpaid labour with another parent, the demands on both are expected to produce similar caregiving and labour supply responses. Supporting the microstructural perspective, single fathers in the USA are more involved in childcare than partnered fathers (Coles, 2015) and have lower employment, avoid working long or unusual hours, and benefit more from childcare availability (Hook & Chalasani, 2008).

Others have suggested that the pressures of family circumstances are moderated by gender (Coles, 2015; Hook & Chalasani, 2008). Partnered fathers may have better opportunities than single fathers (and mothers) for investing in paid work as they can rely on their partners to assume a larger share of childcare and household responsibilities (Killewald & García-Manglano, 2016; Killewald & Gough, 2013). However, increased maternal employment as well as changing gender roles towards more active fathering (Gibb et al., 2014; Koslowski, 2011; Pollmann-Schult & Reynolds, 2017) decrease the fatherhood premium among partnered men (Bergsvik et al., 2020; Mari, 2019), which could reduce the employment difference between single and partnered fathers. Single fathers may also be better able than single mothers to rely on childcare assistance from kin (Hook & Chalasani, 2008). According to Mastrogiacomo et al. (2013), single fathers’ labour supply is more elastic than that of partnered fathers, but lower than that of single mothers. Different views of whether single parenthood affect the employment of fathers and mothers alike thus diminish the chances of forming strong expectations of these differences in the context we study.

### Labour Market and Policy in Finland

The 1990s economic recession caused a major and persistent employment shock in Finland and a restructuring of the labour market towards more highly skilled occupations, which weakened employment opportunities for those with lower levels of education (Asplund & Maliranta, 2006). The 1990s also witnessed an increase in temporary work contracts, particularly among women (Nätti et al., 2005). The aftermath of the 2008 financial crisis had further negative impacts on the labour market, and since then, employment has remained at a lower level than in neighbouring countries (Kyyrä & Pesola, 2020). The weakening labour market for low educated workers has likely contributed to the widening single-parent employment gap as single parenthood at the same time became increasingly associated with low education, as discussed earlier.

Public policies can shape the employment opportunities and incentives of both single and partnered parents. In general, Finnish family policies are characteristic of the Nordic welfare regime that aims to promote gender equality in paid and unpaid labour (Esping-Andersen, 1999). Policies such as parental leaves support mothers’ attachment to the job market and strongly subsidised high-quality childcare services can be especially important. A Finnish specialty in this regard is public childcare that is available also during evenings and night-time, targeted at parents working irregular hours (Moilanen et al. 2019). These policies would be expected to support the employment of low educated single parents in particular and thus reduce the single-parent employment gap. Although findings that less educated parents are less likely to use formal childcare services question this argument, these gaps are small in Finland in European comparison (Pavolini & Van Lancker, 2018).

A peculiarity of Finnish family policies is the popularity of the child home care allowance, which is a subsidy paid after parental leave to parents whose under-three-year-old child is not in municipal day care (Sipilä et al., 2010). This cash-for-care policy was extended to cover all families with children under the age of three in the early 1990s (Hiilamo & Kangas, 2009). Although its value in relation to earnings as well as other benefits has decreased since the mid-1990s, it is considered a key explanation of the comparatively low employment rates of Finnish mothers with young children and argued to weaken women’s labour market position in general (OECD, 2020; Sipilä et al., 2010). Importantly for our study, long parental leaves, which the cash-for-care policy promotes, have particularly negative implications for the employment of single mothers (Morosow & Jalovaara, 2019).

Although Nordic countries’ generous social benefits reduce single-parent poverty (Brady & Burroway, 2012; Maldonado & Nieuwenhuis, 2015), means-tested support such as unemployment and housing benefits and social assistance benefits may discourage employment if additional earnings do not markedly increase the family income. Employment disincentives have in general weakened since the late 1990s as many family benefits as well as unemployment benefits and social assistance did not keep pace with earnings, and income taxation became more favourable to those with labour market earnings (e.g., Honkanen, 2020; Honkanen et al., 2007). Despite these trends, many single parents rely on means-tested benefits such as unemployment and housing benefits and social assistance. In the early 2000s, 40% of single-parent households received housing benefits and 30% received social assistance, respectively, and while these shares decreased over the next decade, by 2015 they had increased again to 50% and 30%, respectively (Social Insurance Institution, 2021; Sotkanet, 2022). These figures are much lower among two-parent households. Because these benefits, as well as the basic unemployment benefit, are affected by labour market earnings, many single parents face severe employment disincentives. Viitamäki (2015) estimated that the effective tax rate upon employment for unemployed single parents is over 70% up to earnings close to the women’s median. Such disincentives are likely to affect the employment of less educated single parents in particular. Kärkkäinen (2011), whose estimations accounted for the sociodemographic profiles of unemployed job seekers, estimated that up to 30% of unemployed single parents faced an effective tax rate over 80%, which was twice that of unemployed partnered parents.

## Summary

We expect that educational divergence in single parenthood has contributed to the growth and persistence of the single-parent employment gap through two reinforcing channels. First, it has led to a compositional change in single parenthood, where single parents have lower average levels of education. The structural changes in Finnish labour markets have favoured the employment of educated workers compared to those with less education, which can have contributed to the single-parent employment gap. Although Finnish social benefit systems have generally been reformed towards fewer employment disincentives, especially during the 2000s, many unemployed workers and single parents in particular face such disincentives. As a result of single parents’ weakened educational profile, these disincentives may affect an increasing share of single parents relative to partnered parents, as suggested by trends in reliance on many means-tested benefits. In conjunction with the structural changes and features of the social benefit system, the compositional change in single parenthood can thus have decreased (average) single parents’ employment opportunities as well as their employment incentives.

Second, we expect that the single-parent employment gap is larger in low than high education groups. Single parents’ labour supply may be more elastic to offered wages and working hours, not least due to their higher childcare demands. Even though the Finnish family policy system offers extensive support to childcare needs, low educated single parents may still struggle to combine childcare and employment, especially if faced with family-unfriendly working hours. Low educated single mothers can be particularly likely to face employment disincentives given the likely wages offered (cf. Kärkkäinen, 2011; Viitamäki, 2015), even though trends in educationally stratified employment disincentives are not clear. Educational divergence has thus made single parenthood more common in educational groups where single parenthood may have the strongest negative employment effect.

We analyse both mothers and fathers. Theoretically, it is not altogether clear whether single parenthood means the same regardless of gender. The labour market and social policy conditions faced by single mothers and fathers are broadly similar, leading to expect that the above pathways work in similar ways for the two groups. However, given women’s concentration in lower-wage occupations, employment disincentives can be more salient among women than men at the same educational level.

## Data and Methods

### Data

We used data formed at Statistics Finland that linked a longitudinal population register and registers of employment, educational degrees, and vital events. The data cover all persons registered in Finland between 1987 and 2018 and include annual information on family type and children living in the household, individual economic activity, and monthly data on completed educational degrees beyond compulsory education. The analytical sample (13,399 thousand person years, men and 14,882 thousand person years, women) was limited to parents aged 18 to 49 years who had at least one child aged 1–17 years (mothers) or 0–17 years of age (fathers) living in the same household. Persons born outside Finland were excluded because information on their educational histories was often deficient. A partnered parent is a parent who has a married or cohabiting partner, or a registered partner in same-sex couples, living in the same household, and a single parent is a parent who is neither married nor co-residing with a partner. Data on cohabiting unions are inferred from data on dwellings and other register data (Jalovaara & Kulu, 2018). Our data do not have information on shared residence or other residence arrangements, and consequently, we cannot distinguish households with different child residence arrangements.

Mothers with children under 1-year-old children were excluded. Finnish family policies allow paid maternity and parental leave until the child is nine months old, and the great majority of mothers use all of this leave. It is difficult to determine the mother’s employment situation during the child’s first year, because in most cases (but not all), the mother’s situation is recorded as her labour market status before the leave.

Employment is a binary variable based whether the individual has an employment contract or is self-employed in the last week of the year. The non-employed include unemployed job seekers, students, pensioners (in practice, persons on disability pension), and others outside the labour force (including homemakers). This measure provides the most detailed information of economic activity in the register. Additional analysis of the months spent in employment during the year shows a strongly bimodal distribution, where the great majority of individuals is employed either for twelve or zero months, which improves the validity of the measure we use. The employment indicator does not distinguish between full-time and part-time workers. Finnish parents work part-time less often than parents in most European countries, and 83% and 68% of employed single and partnered mothers, respectively, work 36 h or more per week (respective shares for fathers are 96% and 93%, respectively) (Sutela, 2015).

Educational attainment measures the person’s highest level of completed education at the end of the year in four categories: lower secondary (9 years, ISCED 0–2), indicating no data on degrees beyond this level; upper secondary (vocational education or academic high school, ISCED 3–4); lower tertiary (post-secondary vocational education or bachelor’s degree, ISCED 5–6); and higher tertiary (master’s degree or higher, ISCED 7–8).

The age of the parent and age of the youngest child are the two other compositional variables. The age of the parent was grouped into three categories: 18–29, 30–39, and 40–49 years. The age of the youngest child in the household was collapsed into three categories 1–2 years (for mothers) and 0–2 years (for fathers), 3–6 years, and 7–17 years, reflecting family policies in Finland and its school system: parents of under-three-year-old children are entitled to home care leave and benefit, and children start school the year they turn seven. Most children from age three to six attend day care, especially if they do not have younger siblings. We used the age of the youngest child instead of the number of children as the compositional variable because it has a stronger independent effect on Finnish mothers’ employment than the number of children (Statistics Finland, 2014).

In the decomposition analysis, we compared seven periods from 1987 to 2018: 1987–1990, 1991–1994, 1995–1998, 1999–2003, 2004–2008, 2009–2013, and 2014–2018. These time periods reflect macroeconomic conditions and labour market trends. The study covers periods of economic upswings and increasing employment (1987–1990, 1995–1998, and 2014–2018), recessions and decreasing employment (1991–1994 and 2009–2013), and periods of stable employment (1999–2003 and 2004–2008).

Table 1 shows the distributions of observations (in person-years) across the compositional variables in the first and last periods for partnered and single mothers and fathers.

**Table 1:** Distributions of partnered and single mothers and fathers (person years) by the compositional variables in 1987–1990 and 2014–2018, by percentage

	1987–1990		2014–2018		1987–1990		2014–2018	
	Single mothers	Partnered mothers	Single mothers	Partnered mothers	Single fathers	Partnered fathers	Single fathers	Partnered fathers
*Educational attainment*								
Lower secondary	37	31	13	5	41	31	15	9
Upper secondary	43	42	48	38	40	40	54	50
Lower tertiary	17	22	26	35	14	21	20	24
Higher tertiary	4	5	12	22	5	8	10	17
Total	100	100	100	100	100	100	100	100
*Age*								
18–29 yrs	18	15	14	10	7	13	4	9
30–39 yrs	48	51	38	44	43	48	32	43
40–49 yrs	34	34	47	46	51	39	64	47
Total	100	100	100	100	100	100	100	100
*Age of the youngest child*								
1–2 yrs*	11	20	12	23	4	30	4	34
3–6 yrs	25	28	28	30	16	26	20	28
7–17 yrs	64	52	59	47	80	44	76	39
Total	100	100	100	100	100	100	100	100
N (person years)	268,904	1,887,994	372,932	1,581,324	35,749	2,019,420	56,331	1,631,512

^*^Age of the youngest child included 0-year-old children for fathers (e.g. first category 0–2 years)

### Method

We used the CS decomposition method (Chevan & Sutherland, 2009) separately for mothers and fathers. Here, we provide a summary of the method, and a more detailed and technical description can be found in Appendix.

The CS method is an extension of Das Gupta’s (1993), or DG, decomposition method. DG decomposes the employment rate difference between partnered and single parents into the composition effects of education, the age of the parent, the age of the youngest child, and the rate effect.

Composition effects tell what shares of the gap in employment can be attributed to compositional differences in the background factors. CS additionally decomposes the composition effects between the categories of each variable, which indicates where in the distribution of the variable the differences are the most consequential. The differences in the shares of partnered and single mothers in each category are weighted by a function of the relative size of the category and the average employment rates in it. For example, the above discussion suggested that educational differences are likely the largest at the ends of the distribution: partnered parents are more likely than single parents to have higher tertiary education (where employment rates are high), and the opposite holds for lower secondary education. In such a case, the category composition effect of higher tertiary education is positive and the respective effect for lower secondary education is negative.

In DG, the rate effect summarises the average difference in employment rates between partnered and single parents after adjusting for their compositional differences. The rate effect can be interpreted as reflecting differences in labour supply or demand between the groups, or differences in unmeasured background factors.

CS further estimates rate effects for each category of the background variable. The overall rate effect in DG is divided equally between the background variables, so that the sums of the category rate effects are the same for each variable. This way, the category rate effect estimates can be interpreted as indicating the importance of a difference in the rate effect in one category as compared to the other categories of that variable. The category rate effects are thus similar to a decomposition of interaction effects.

The importance of each category depends not only on the standardised difference in employment rates in the category but also on the size of that category. For example, a large category A can have a bigger rate effect than small category B even if the standardised difference in employment rates are the same. The size of category A gives it more weight, and it thus makes a bigger contribution to the overall single-parent employment gap.

The CS method provides additional insight into the sources of the single-parent employment gap. Next to explaining how much of it is due to compositional differences by each background variable, the method reveals where in the distribution of these variables the differences are the most important. It also identifies the subgroups in which the employment rate differences are the most consequential.

## Results

### Shifts in Sociodemographic Profiles

Table 1 shows the education, age-group, and age of the youngest child distributions among partnered and single mothers and fathers in 1987–1990 and in 2014–2018. The distributions for each period are presented in Supplementary Tables S1 and S2.

In 1987–1990, single and partnered mothers were relatively similar in terms of age and educational attainment. Single mothers were more likely than partnered mothers to have school-aged children, reflecting that single motherhood results primarily from partnership dissolution. The differences between single and partnered fathers were larger during the first period. Single fathers were clearly older than partnered fathers, and the majority of single fathers had school-aged children. Single fathers were, on average, less educated than partnered fathers, and they were more likely to have only lower secondary level education and less likely to have tertiary-level education.

Educational backgrounds showed the largest change between 1987–1990 and 2014–2018. Reflecting the widening educational gradients in single parenthood (Härkönen, 2017; McLanahan, 2004), single parenthood became increasingly characterised by lower educational attainment, especially among mothers. The changes in the age of the parents and the age of the youngest child were smaller. The age gap between single and partnered fathers grew somewhat, whereas a slightly larger share of single fathers had children of preschool age (offset by a smaller share of single fathers with school-aged children). There were fewer changes between single- and partnered mothers.

### Trends in Employment Rates

**Fig. 2:**
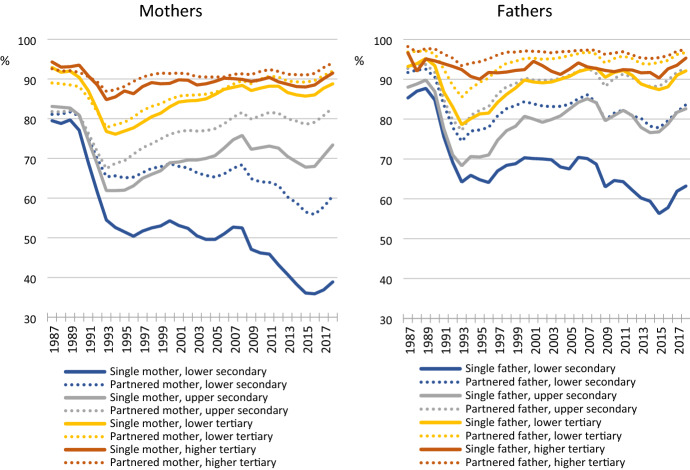
Employment rates (%) of single and partnered parents, by educational attainment, 1987–2018

Figure 2 shows trends in partnered and single parents’ employment rates by educational attainment. These numbers in tabular format, as well as corresponding trends according to the other background variables, are included in the Supplement Tables S3 and S4.

In the late 1980s, employment rates were close to or above 80% in all educational groups and nearly 100% among highly educated fathers. In the late 1980s, single mothers had high employment rates and notably, in all educational groups but the lowest, single mothers’ employment rates exceeded those of partnered mothers by 1–2 percentage points. Single fathers, on the other hand, had employment rates that were about 5 percentage points lower than those of partnered fathers in most sub-groups.

Employment rates declined in all groups during the 1991–1994 economic recession. From then onwards, single parents have had lower employment rates than partnered parents in all educational groups. Yet the trends in the single-parent employment gap have been clearly stratified by education. The gaps have been small especially among tertiary educated mothers, ranging between 1 and 2 percentage points, with a slight increase since the 2008 financial crisis. Interestingly, the gap has been wider among tertiary educated fathers—largely between 4–6 percentage points—although fathers have had higher employment rates than mothers in both groups.

The employment gaps have been clearly larger among the secondary educated. Among upper secondary educated mothers, the employment gap was in single mothers’ favour in the late 1980s, grew to 4.5 percentage points in the early 1990s, remained between 6 and 8 percentage points and finally grew to 10.3 percentage points in the last period. Single fathers had lower employment rates than partnered fathers already in the 1980s, but this gap grew during the recession and has remained between 8 and 11 percentage points since. The largest single-parent employment gaps are found in the lower secondary education group. Among mothers, the originally small gap grew to 10 percentage points in 1991–1994 and remained around 15 percentage points till the 2008 economic crisis, after which it widened to again to end up at 20 percentage points in the last period, during which lower secondary educated single mothers’ employment rate fell below 40%, to half of its level from 25 years earlier. Among fathers, the gap grew more steadily to 20 percentage points. Even though the gaps ended up of similar size among mothers and fathers in this group, lower secondary educated single fathers have clearly higher employment rates than mothers in the same educational group.

Trends in the single-parent employment gap have thus been strongly stratified by education. The gap grew during the 1990s economic crisis, and especially among (lower) secondary educated mothers, in the aftermath of the 2008 crisis. The trends do not follow any obvious patterns in the development of the social security system. Rather, they may reflect how social security interacts with economic cycles and structural changes.

Briefly commenting on the trends by the other background variables (see Supplement), the largest single-parent employment gaps were among parents with small children, with single mothers with small children having the lowest employment rates. By age, the largest single-parent employment gaps were in the youngest age group.

### Decomposition Analysis

The last section showed how the single-parent employment gap has developed in different population subgroups. Because the relative sizes of these groups have changed, we next analyse the contributions of the change in sociodemographic composition and the changes in employment gaps to the overall employment gap.

**Fig. 3:**
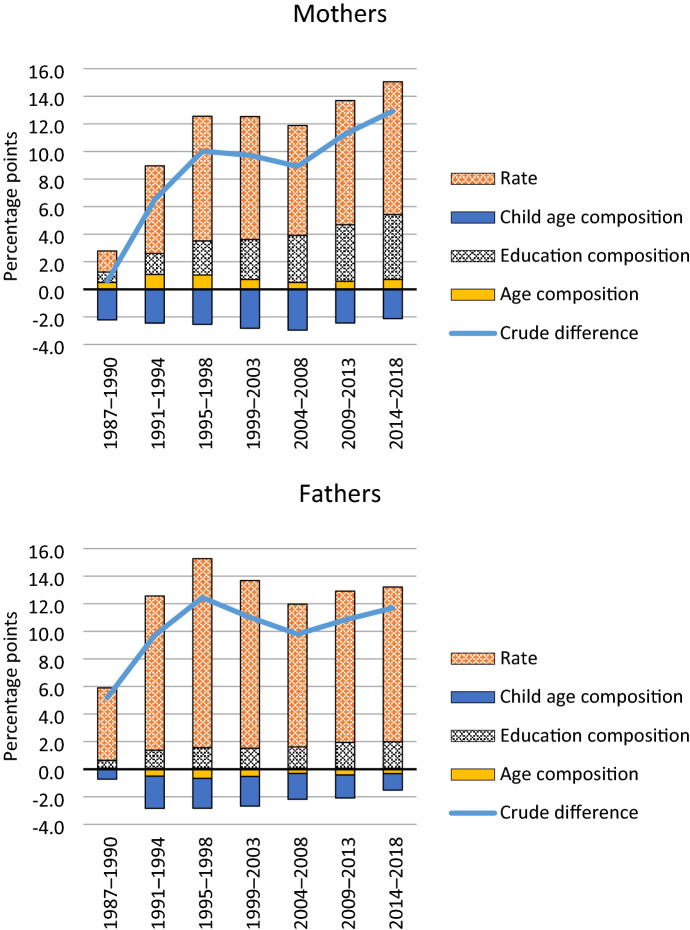
The crude single-parent employment gap, composition effects by background variable, and the rate effect for mothers and fathers

Figure 3 first presents results from the decomposition using Das Gupta’s (1993) method. The lines show the crude single-parent employment gaps, which among mothers increased from nil in 1987–1990 to 10% points in 1995–1998 and further to 13% points in 2014–2018. The single-father employment gap grew from 5% points in 1987–1990 to 12% points in 1995–1998, diminished in the next ten years, and reached 12% points again in 2014–2018.

The majority of the widening single-mother employment gap and almost all of the increased single father employment gap can be attributed to an increase in the rate effect. The rate effect increased during the early-to-mid-1990s and stayed relatively stable since (see also Tables S5 and S6 in the Supplement). This corresponds to the descriptive analysis of the employment gaps above. The rate effect has been larger throughout the follow-up among fathers than mothers (9.6 p.p. and 11.2 p.p., respectively, in the last period).

Among mothers, the increasing educational composition effect contributed steadily to the growing single-mother employment gap and in the last three periods, 36% (4.7 p.p. / 12.9 p.p.) of the crude single-mother employment gap could be attributed to the educational composition effect. The educational composition effect was less important for fathers, and 17% of the crude employment gap in the latest period (2.0 p.p. / 11.7 p.p.) was attributable to it. Single mothers’ age composition contributed to their employment gap (positive effect) and single fathers’ age composition reduced their employment gap (negative effect), although in both cases the effects were small. Single parents’ children are older than those of partnered parents, which reduced the employment gaps.

Table 2 presents the results from the CS decomposition into category composition and category rate effects by education for mothers and Table 3 for fathers. The results for all of the background variables are shown in Supplementary Tables S5 and S6.

**Table 2:** Category composition and rate effects for education from the Chevan–Sutherland decomposition 1987–1990 to 2014–2018, percentage points, mothers

	1987–1990	1991–1994	1995–1998	1999–2003	2004–2008	2009–2013	2014–2018
*Employment rate, %*							
Single mothers	83.0	67.7	65.0	70.1	73.7	73.6	72.3
Partnered mothers	83.6	74.2	75.0	79.8	82.6	84.9	85.2
Crude difference (%-points)	0.6	6.5	10.0	9.7	8.9	11.3	12.9
Total for rate	1.5	6.4	9.0	8.9	8.0	9.0	9.6
Total for composition	− 1.0	0.2	1.0	0.8	1.0	2.2	3.3
*Composition*							
Educational attainment-total	0.7	1.5	2.5	2.9	3.4	4.1	4.7
Lower secondary	− 3.5	− 3.9	− 4.9	− 5.4	− 5.4	− 4.8	− 3.8
Upper secondary	− 0.9	− 0.6	− 0.9	− 1.9	− 3.5	− 5.6	− 7.5
Lower tertiary	4.5	4.7	5.9	6.8	7.4	7.8	7.5
Higher tertiary	0.7	1.3	2.4	3.5	4.9	6.6	8.5
*Rate*							
Educational attainment							
Lower secondary	0.3	0.8	0.9	0.8	0.7	0.7	0.6
Upper secondary	0.2	1.0	1.5	1.5	1.3	1.6	1.8
Lower tertiary	0.0	0.3	0.5	0.5	0.4	0.5	0.6
Higher tertiary	0.0	0.1	0.1	0.1	0.2	0.2	0.3

**Table 3:** Category composition and rate effects for education from the Chevan–Sutherland decomposition 1987–1990 to 2014–2018, percentage points, fathers

	1987–1990	1991–1994	1995–1998	1999–2003	2004–2008	2009–2013	2014–2018
*Employment rate, %*							
Single fathers	88.6	73.3	73.9	79.7	82.6	80.1	79.7
Partnered fathers	93.7	83.0	86.3	90.8	92.4	91.0	91.4
Crude difference (%-points)	5.2	9.7	12.4	11.0	9.8	10.8	11.7
Total for rate	5.3	11.2	13.7	12.2	10.4	11.0	11.2
Total for composition	− 0.1	− 1.5	− 1.3	− 1.2	− 0.6	− 0.1	0.5
*Composition*							
Educational attainment-total	0.6	1.4	1.6	1.5	1.6	1.9	2.0
Lower secondary	− 6.4	− 4.8	− 5.0	− 5.3	− 5.2	− 5.1	− 4.3
Upper secondary	− 1.7	− 2.0	− 2.1	− 2.7	− 3.6	− 3.7	− 4.7
Lower tertiary	5.8	5.0	5.1	5.5	5.4	5.1	4.6
Higher tertiary	2.9	3.3	3.5	4.1	5.0	5.7	6.3
*Rate*							
Educational attainment							
Lower secondary	0.8	1.3	1.4	1.2	1.1	1.1	0.9
Upper secondary	0.7	1.8	2.2	1.9	1.6	1.9	2.1
Lower tertiary	0.2	0.6	0.7	0.6	0.5	0.4	0.5
Higher tertiary	0.1	0.1	0.2	0.3	0.3	0.3	0.2

We first consider the category composition effects among mothers and find both positive and negative composition effects, as is standard when decomposing between categories. Both the positive and negative category effects grew over time, reflecting the evolving educational divergence in single motherhood. However, the positive effects of tertiary education grew more. In particular, the effect of higher tertiary education increased steadily from 0.7 to 8.5 percentage points, and that of lower tertiary education from 4.5 to 7.5 percentage points. At the same time, the category composition effects of lower secondary education changed less and were finally of similar size in the last period as in the 1990s.

The category composition effects are the differences in the shares of partnered and single mothers in each category, weighted by a function of the size of that category and the average employment rate in that category. The contribution of the increasing difference in the shares of partnered and single mothers with tertiary education is a function of their high employment rates and increasing size, whereas the decreasing size and employment rates in the lower secondary education category have kept its category rate effect at check. The growing gap in the shares of single and partnered mothers who have tertiary, and especially higher tertiary education, has thus become increasingly important.

The total rate effect for each period (see Fig. 3) is divided equally among the three background variables (see Supplementary Tables S5 and S6). Consequently, the rate effect of education is one-third of the total rate effect. The decomposition of the rate effect by educational categories shows in which groups the employment difference between partnered and single mothers matters the most for the crude single-mother employment gap.

The category rate effects reflect the standardised employment gaps within each category, weighted by the size of that category. From the early-1990s onward, upper secondary education had the largest and growing category rate effect. Even without major changes in the employment gaps among the upper secondary educated since the 1990s (see Sect. 4.3), the size of this group has meant a large contribution to the overall gap. In other words, the single-mother employment gap has grown partly because more single mothers (compared to partnered mothers) have upper secondary education, a category in which single mothers are less employed than partnered mothers. The importance of the size of the group becomes visible also when compared to the lower secondary educated. This group has witnessed an increase in the employment gap but a decrease in size, leading to a stable category rate effect since the early 1990s, which if anything has decreased since the early 2000s.

Likewise, despite the relatively small employment gap in the lower tertiary education category, the growing size of this group means that its rate effect contributed in the last period as much to the overall gap as that of lower secondary education. However, the contribution of the higher tertiary education rate effect was limited, reflecting the small employment rate differences in that group.

For fathers, the strengthening of the educational composition effect resulted especially from higher tertiary-level educated fathers. Due to their declining numbers, fathers with only lower secondary education contributed less to the single father employment gap in the last compared to the earlier periods, while the role of fathers with upper secondary level education has increased. Similar to the category rate effects among mothers, fathers with upper secondary level education have, since 2009, had the largest rate effect contribution to the employment gap.

The other category composition and rate effects are presented in Supplementary Tables S5 and S6. By age, the largest category rate effects for mothers are in the 30 − 39-year-old group, and increasingly, in the 40 − 49-year-old group, and for fathers, in the age group of 40–49 years old. By age of the youngest child, the largest category rate effects are in the group of mothers or fathers with school-aged children. Importantly, the category rate effect of mothers with 1-to 2-year-old children is relatively limited. Despite the large difference in employment rates in this group, the small size of this group means that its contribution to the single-mother employment gap is modest.

The decompositions are additive, meaning that we can estimate how much the category composition effect and different rate effects have together contributed to the changes in single-parent employment gaps. Thirty-three percent of the increase in the single-mother employment gap from 0.6 to 12.9 percentage points from 1987–90 to 2014–18 could be attributed to the change in the educational composition effect (from 0.7 p.p. to 4.7 p.p.). For fathers, 22% of the increase in the employment gap ((2.0 p.p.–0.6 p.p.) / (11.7 p.p.–5.2 p.p.)) was attributable to the change in the educational composition.

We can likewise estimate how much the change in the category rate effects contributed to the change in the overall gaps. Attention is drawn to the two lowest educational categories, where single parenthood grew the most and the employment gaps were the largest. The rate effect among mothers with lower secondary education increased from 0.3 to 0.6 percentage points, and in the upper secondary education category from 0.2 to 1.8 percentage points. Together, 15% ((0.6 − 0.3) + (1.8 − 0.2)) / 12.3) of the growth in the single-mother employment gap can be attributed to the increasing rate effect in these two educational categories. The respective contribution of the changes in the rate effects in the two lowest educated groups of fathers was 23% ((0.9 − 0.8) + (2.1 − 0.7)) / 6.5).

Together, the changes in the educational composition effect and in the rate effects in the two lowest educational level groups accounted for 48% (33% + 15%) of the increase in the single-mother employment gap, and for 45% (22% + 23%) of the increase in the single father employment gap. The contribution of these two changes was of similar magnitude, although the sources of the changes differed between mothers and fathers. The remaining gap is due to category rate effects among the tertiary educated and more importantly, composition and rate effects in the two other compositional variables.

## Discussion

The widening gap between employment rates of Finnish single and partnered parents has gone unnoticed among researchers and policy-makers. In the late 1980s, single fathers had almost as high employment rates as partnered fathers, and single mothers’ employment was on par with that of partnered mothers. Single parents’ employment was particularly hard hit by the 1990s economic crisis and has remained lower than that of partnered parents, and in 2018, the employment rate of single mothers was 12 percentage points lower than that of partnered mothers, and the respective gap among fathers was 11 percentage points. This questions the Finnish welfare model’s capacity to support employment among all parents, which has been considered its central feature (e.g., Esping-Andersen, 1999).

The objective of this study was to analyse the role of social demographic change in understanding the changing single parenthood employment gaps in Finland. We focused on the relative weakening of single parents’ educational backgrounds due to an increase in single parenthood particularly among those with lower levels of education. Educational divergence in family demography has attracted major attention (McLanahan, 2004; McLanahan & Jacobsen, 2015), and our results are among the first to quantify the inequality consequences of uneven family change (cf. Bernardi & Boertien, 2017; Härkönen, 2018; Zagel, et al., 2021).

Educational divergence has created a double disadvantage for single parents’ employment. First, it has gradually shifted the educational profiles of single parents towards educational groups which have lower employment rates in general. One-third of the increase in the employment gap between single and partnered mothers, and about one-fifth of the increased gap among fathers, can be attributed to this compositional change.

Second, the growth of single-parent employment gap has been stratified by education. The gap remained small among tertiary-level educated parents (and mothers in particular) but has been large and persistent among lower and upper secondary educated parents since the 1990s recession, possibly due to a combination of labour market structural changes and employment disincentives that have had a detrimental effect on the employment of single parents with no more than secondary education. Notably, the weaker employment situation of single parents concerns not only the lowest educated group—where the gap between single and partnered parents has clearly grown—but also men and women with upper secondary level education, who constitute about half of the study population. Educational divergence thus also shifted single parents’ educational profiles towards groups where single-parent employment gaps have been the largest since the early 1990s. Altogether, almost half of the change in the overall single-parent employment gap could be attributed to the increase in single parenthood among the low educated.

A detailed analysis of the factors that have hurt the employment of less-educated single parents was beyond the scope of this study and is left for future research. Possible reasons include interactions of the social benefit system with changes in the labour market. Increased skills demands together with highly centralised wage bargaining, which increases wage rigidity, may have reduced the demand for less-skilled workers (Kalleberg & Vallas, 2017; Kanninen & Böckerman, 2013). At the same time, temporary work contracts (Nätti et al., 2005) and non-standard and inflexible working hours (Janzen & Kelly, 2012; Moilanen et al., 2019) have increased. These changes may have hurt less educated single parents in particular. Single parents can have less flexibility in accepting non-standard working hours. They may also have higher employment disincentives due to social benefits, which can disincentivise the take-up of low-to-middle wage employment and especially ones with temporary contracts or reduced hours.

National debates have in particular pointed to widely used means-tested benefits such as the unemployment benefit, housing benefits and social assistance that create a high effective tax rate on work for earnings up to the women’s median (Kärkkäinen, 2011; Viitamäki, 2015). Together with less flexibility in working conditions, these disincentives can reduce lower-educated single parents’ labour supply, a pattern which corresponds with our results. The gradual shift of single parenthood towards lower educational groups has meant that employment disincentives can affect a growing share of single parents. Therefore, even if social policies in general have been reformed towards incentivising paid work (Honkanen, 2020; Honkanen et al., 2007), the compositional change in single parenthood can have partly or fully offset the effects of these policies.

Cash-for-care is another policy that has been argued to disincentivise mothers’ employment (OECD, 2020; Sipilä et al., 2010). However, according to our results this policy has had at most a minor effect on the single-parent employment gap. Only parents with children under the age of three are eligible for the benefit. Although the single-parent employment gap in this group was large, due to its small size its contribution to the overall single-parent employment gap is small. Long family leaves can nevertheless contribute to the employment gap over the life course, as they can have negative effects on later employment and earnings of single mothers in particular (Morosow & Jalovaara, 2019).

The inclusion of single fathers was a novel feature of our analysis. Although single fatherhood remains relatively rare, it has increased in many Western countries (Eggebeen et al., 2012; Nieuwenhuis, 2020). Similar to single motherhood, single fatherhood is increasingly concentrated in the lower educated groups. Importantly, the employment trends of single fathers followed those of single mothers, both at the aggregate level and in different educational groups. These findings are in line with microstructural theories of single parenthood, which highlight the specific circumstances faced by single parents regardless of gender (Coles, 2015; Hook & Chalasani, 2008). However, even though the single parenthood employment gap is similar among fathers and mothers, fathers have consistently had higher employment rates than mothers, reflecting persistent gendered patterns in parents’ employment. Another gender difference concerned the sources of the single-parent employment gap. Maybe surprisingly, the rate effect was higher among fathers than mothers and its increase accounted for the change in the single father employment gap. The larger rate effect can reflect gender differences in the effects of single parenthood on employment, or differences in unmeasured characteristics (such as reasons for resuming main caring responsibility). All in all the analysis suggests that we should problematise theoretical approaches to single parenthood, which typically focus on single mothers, and invite future research into single fathers.

A limitation of our analysis is that the data do not allow us to distinguish between single parents with different child residence arrangements. Shared residential custody arrangements have increased in Finland, especially among the highly educated (Miettinen et al., 2020). Future research can inquire how shared residence shaped single parents’ employment in different educational groups.

Our analysis speaks to the unequalising potential of family bifurcation. It also points to the policy challenges in effectively responding to family bifurcation, which can create double disadvantages in the intersection between low education and single parenthood and where traditional policies of childcare promotion and income transfers may not be enough to support single-parent employment, even if they can keep their poverty rates at low levels (Brady & Burroway, 2012; Maldonado & Nieuwenhuis, 2015). Future research should more closely analyse the combination of labour market structural changes and social policies which may have depressed single parents’ employment.

### Electronic supplementary material

Below is the link to the electronic supplementary material.

Supplementary file1 (PDF 273 KB)

## The Chevan–Sutherland decomposition

Starting from Das Gupta (DG, 1993) and using lowercase letters to denote partnered parents and uppercase letters to denote single parents, the difference in employment rates between the groups can be decomposed into three composition effects (of education [*I*], age [*J*], and age of the youngest child [*K*]) and the rate effect (*R*):

1 $$t_{ \ldots } - T_{ \ldots } = \left[ {R\left( {\overline{t}} \right) - R\left( {\overline{T}} \right)} \right] + \left[ {I\left( {\overline{a}} \right) - I\left( {\overline{A}} \right)} \right] + \left[ {J\left( {\overline{b}} \right) - J\left( {\overline{B}} \right)} \right] + \left[ {K\left( {\overline{c}} \right) - K\left( {\overline{C}} \right)} \right]$$

These effects are differences in the standardised (for the other terms) rates between partnered and single parents (see Das Gupta, 1993, p. 63–66).

The Chevan–Sutherland method (CS, 2009) estimates additional category effects. Category composition effects are estimated as the group difference in the standardised rates for each category of the variable. For each group, the standardised rate for each variable is the sum of the standardised rates for each category. The *I*, *J*, and *K* –effects are thus

2 $$I\left( {\overline{a}} \right) - I\left( {\overline{A}} \right) = \mathop \sum \limits_{i..} I\left( {\overline{a}} \right)_{i..} - \mathop \sum \limits_{i..} I\left( {\overline{A}} \right)_{i..}$$

3 $$J\left( {\overline{b}} \right) - I\left( {\overline{B}} \right) = \mathop \sum \limits_{.j.} J\left( {\overline{b}} \right)_{.j.} - \mathop \sum \limits_{.j.} J\left( {\overline{B}} \right)_{.j.}$$

and

4 $$K\left( {\overline{c}} \right) - K\left( {\overline{C}} \right) = \mathop \sum \limits_{..k} K\left( {\overline{c}} \right)_{..k} - \mathop \sum \limits_{..k} K\left( {\overline{C}} \right)_{..k}$$

Because a group difference in the size of one category must be offset by a difference of the opposite sign in at least one other category, there are generally both positive and negative category composition effects (CS, p. 435).

In DG, the rate effect applies equally to all background variables. CS estimates additional category rate effects. In the three-factor case, single mothers’ standardised rates of categories for the variables *I*, *J*, and *K* are

5 $$R\left( {\overline{T}} \right)_{i..} = \mathop \sum \limits_{{{\text{jk}}}} \frac{{\frac{{n_{{{\text{ijk}}}} }}{{n_{ \ldots } }} + \frac{{N_{{{\text{ijk}}}} }}{{N_{ \ldots } }}}}{2}T_{{{\text{ijk}}}} \frac{1}{{{\text{NV}}}}$$

6 $$R\left( {\overline{T}} \right)_{.j.} = \mathop \sum \limits_{{{\text{ik}}}} \frac{{\frac{{n_{{{\text{ijk}}}} }}{{n_{ \ldots } }} + \frac{{N_{{{\text{ijk}}}} }}{{N_{ \ldots } }}}}{2}T_{{{\text{ijk}}}} \frac{1}{{{\text{NV}}}}$$

7 $$R\left( {\overline{T}} \right)_{..k} = \mathop \sum \limits_{{{\text{ij}}}} \frac{{\frac{{n_{{{\text{ijk}}}} }}{{n_{ \ldots } }} + \frac{{N_{{{\text{ijk}}}} }}{{N_{ \ldots } }}}}{2}T_{{{\text{ijk}}}} \frac{1}{{{\text{NV}}}}$$

and similarly for partnered parents when *T* is replaced by *t.* Because the rate effect applies equally to each background variable, the contribution of each category is obtained by scaling it by the reciprocal of the number of background variables (NV), and the sums of the standardised category rates are equal for each variable (CS, p. 432). For single parents:

8 $$\mathop \sum \limits_{i} R\left( {\overline{T}} \right)_{i..} = \mathop \sum \limits_{j} R\left( {\overline{T}} \right)_{.j.} = \mathop \sum \limits_{k} R\left( {\overline{T}} \right)_{..k}$$

and similarly for partnered parents when *T* is replaced by *t.*

The category composition and rate effects are additive, and their sum equals the crude single parent employment gap:

9 $$\begin{gathered} t_{ \ldots } - T_{ \ldots } = \left( {\mathop \sum \limits_{i..} I\left( {\overline{a}} \right)_{i..} + \mathop \sum \limits_{i..} R\left( {\overline{t}} \right)_{i..} + \mathop \sum \limits_{.j.} J\left( {\overline{b}} \right)_{.j.} + \mathop \sum \limits_{.j.} R\left( {\overline{t}} \right)_{.j.} + \mathop \sum \limits_{..k} K\left( {\overline{c}} \right)_{..k} + \mathop \sum \limits_{..k} R\left( {\overline{t}} \right)_{..k} } \right) \hfill \\ - \left( {\mathop \sum \limits_{i..} I\left( {\overline{A}} \right)_{i..} + \mathop \sum \limits_{i..} R\left( {\overline{T}} \right)_{i..} + \mathop \sum \limits_{.j.} J\left( {\overline{B}} \right)_{.j.} + \mathop \sum \limits_{.j.} R\left( {\overline{T}} \right)_{.j.} + \mathop \sum \limits_{..k} K\left( {\overline{C}} \right)_{..k} + \mathop \sum \limits_{..k} R\left( {\overline{T}} \right)_{..k} } \right) \hfill \\ \end{gathered}$$

which was the equation we used in our decompositions.
