# Supplementary material for: Double Disadvantage in a Nordic Welfare State: A Demographic Analysis of the Single-Parent Employment Gap in Finland, 1987–2018
Source: Eur J Popul. 2023 Feb 21;39(1):2. doi: 10.1007/s10680-023-09651-w (PMC9944225; doi:10.1007/s10680-023-09651-w)

Supplementary Tables

Table S1. Distributions of single and partnered mothers (person years) by age, age of the youngest child and educational attainment (%).

		1987–1990	1991–1994	1995–1998	1999–2003	2004–2008	2009–2013	2014–2018
Single mothers, % of all mothers		12.5	14.6	17.0	18.1	18.4	18.3	19.1
Partnered mothers, % of all mothers		87.5	85.4	83.0	81.9	81.6	81.7	80.9
Single mothers, person years (N)		268,904	311,518	344,350	425,198	403,120	379,198	372,932
Partnered mothers, person years (N)		1,887,994	1,817,655	1,685,843	1,921,448	1,793,150	1,689,586	1,581,324
Age, years								
Single mothers	18–29	18.0	16.4	14.4	12.9	13.6	14.7	14.2
	30–39	47.7	44.9	44.2	41.8	35.2	34.4	38.4
	40–49	34.4	38.7	41.5	45.3	51.2	50.9	47.4
		100	100	100	100	100	100	100
Partnered mothers	18–29	15.0	12.6	11.0	10.2	10.6	10.6	9.7
	30–39	51.0	47.4	46.2	44.9	41.4	42.1	44.4
	40–49	34.0	40.1	42.8	44.9	48.1	47.3	45.9
		100	100	100	100	100	100	100
Age of the youngest child, years								
Single mothers	1–2	10.7	12.6	12.1	11.3	11.4	12.5	12.4
	3–6	25.1	24.6	26.7	25.6	24.1	25.8	28.3
	7–17	64.2	62.8	61.2	63.1	64.6	61.8	59.3
		100	100	100	100	100	100	100
Partnered mothers	1–2	20.2	21.0	21.1	20.6	22.0	23.7	23.1
	3–6	28.3	25.9	27.2	27.6	26.5	27.7	29.8
	7–17	51.5	53.2	51.6	51.8	51.6	48.6	47.1
		100	100	100	100	100	100	100
Educational attainment								
Single mothers	Lower secondary	37.0	31.9	27.7	22.8	18.9	16.2	13.3
	Upper secondary	42.7	44.7	45.0	45.9	46.8	47.1	48.4
	Lower tertiary	16.7	19.2	22.4	25.3	26.5	26.7	26.1
	Higher tertiary	3.6	4.2	4.8	6.1	7.8	10.0	12.2
		100	100	100	100	100	100	100
Partnered mothers	Lower secondary	31.5	24.4	18.0	12.8	9.0	6.7	4.5
	Upper secondary	41.6	43.8	43.6	42.8	41.5	39.2	37.8
	Lower tertiary	22.3	25.8	30.5	34.0	35.6	36.0	35.1
	Higher tertiary	4.6	6.0	7.9	10.4	14.0	18.1	22.3
		100	100	100	100	100	100	100

Table S2. Distributions of single and partnered fathers (person years) by age, age of the youngest child and educational attainment (%).

		1987–1990	1991–1994	1995–1998	1999–2003	2004–2008	2009–2013	2014–2018
Single fathers, % of all fathers		1.7	2.1	2.5	2.7	2.8	3.0	3.4
Partnered fathers, % of all fathers		98.3	97.9	97.5	97.3	97.2	97.0	96.7
Single fathers, person years (N)		35,749	42,486	45,988	56,661	54,977	54,331	56,471
Partnered fathers, person years (N)		2,019,420	1,945,653	1,779,871	2,010,003	1,885,394	1,780,370	1,631,512
Age, years		100.0	100.0	100.0	100.0	100.0	100.0	100.1
Single fathers	18–29	6.5	4.7	3.4	3.1	3.8	4.6	4.4
	30–39	43.1	37.6	34.1	31.1	26.4	27.7	32.0
	40–49	50.5	57.7	62.5	65.8	69.9	67.7	63.7
		100	100	100	100	100	100	100
Partnered fathers	18–29	12.9	11.6	10.2	9.8	10.6	10.7	9.4
	30–39	48.4	44.3	43.7	42.9	40.3	41.6	43.4
	40–49	38.7	44.1	46.1	47.2	49.0	47.8	47.3
		100	100	100	100	100	100	100
Age of the youngest child, years								
Single fathers	0–2	4.1	3.4	3.3	3.3	3.6	3.9	4.0
	3–6	16.2	14.7	14.6	14.4	14.4	17.6	19.6
	7–17	79.7	81.9	82.0	82.3	82.0	78.5	76.4
		100	100	100	100	100	100	100
Partnered fathers	0–2	29.9	31.1	31.2	31.2	33.4	35.6	34.0
	3–6	25.9	23.6	25.1	25.6	24.3	25.2	27.5
	7–17	44.3	45.3	43.7	43.2	42.3	39.2	38.5
		100	100	100	100	100	100	100
Educational attainment								
Single fathers	Lower secondary	40.5	34.7	29.9	24.8	20.9	18.6	15.3
	Upper secondary	40.3	44.5	47.5	50.5	52.8	53.1	54.3
	Lower tertiary	14.4	15.7	17.1	18.3	19.1	19.7	20.1
	Higher tertiary	4.8	5.2	5.6	6.4	7.2	8.6	10.2
		100	100	100	100	100	100	100
Partnered fathers	Lower secondary	31.2	26.2	21.5	17.3	14.0	11.5	9.1
	Upper secondary	40.1	43.2	45.3	47.3	48.4	48.9	49.5
	Lower tertiary	20.5	21.7	23.2	24.2	24.5	24.6	24.3
	Higher tertiary	8.1	9.0	10.0	11.3	13.1	15.1	17.1
		100	100	100	100	100	100	100

Table S3. Employment rates (%) by educational attainment, age, and age of the youngest child in 1987–2018, single and partnered mothers

	1987–1990	1991–1994	1995–1998	1999–2003	2004–2008	2009–2013	2014–2018
Single mothers							
Total	83.0	67.7	65.0	70.1	73.7	73.6	72.3
Educational attainment							
Lower secondary	78.8	59.3	51.5	52.7	51.0	44.8	37.2
Upper secondary	82.4	66.5	64.0	68.8	72.7	72.3	69.8
Lower tertiary	91.7	80.2	78.6	83.5	86.9	87.5	86.9
Higher tertiary	93.5	87.2	87.7	89.2	89.7	89.5	89.3
Age, years							
18–29	69.7	43.5	38.0	42.8	47.6	48.6	48.1
30–39	84.7	68.9	65.1	69.7	73.0	72.4	71.1
40–49	87.5	76.6	74.2	78.3	81.1	81.6	80.6
Age of the youngest child, years							
1–2	61.9	39.6	36.6	39.3	43.7	47.5	48.5
3–6	80.7	62.2	59.8	65.7	68.5	68.6	68.1
7–17	87.4	75.5	72.8	77.4	80.9	81.0	79.3
Partnered mothers							
Total	83.6	74.2	75.0	79.8	82.6	84.9	85.2
Educational attainment							
Lower secondary	81.2	69.4	65.9	67.8	66.5	63.5	57.8
Upper secondary	81.7	71.1	71.7	76.3	79.0	80.7	80.1
Lower tertiary	88.6	80.7	81.2	85.3	87.7	89.9	90.2
Higher tertiary	92.1	88.4	89.8	91.2	90.8	91.7	92.1
Age, years							
18–29	70.5	51.7	49.0	54.7	60.5	65.4	65.6
30–39	84.2	73.8	74.4	78.8	81.2	84.2	84.5
40–49	88.4	81.7	82.3	86.5	88.7	89.8	90.0
Age of the youngest child, years							
1–2	68.2	54.8	54.9	58.8	63.8	71.8	73.2
3–6	83.5	73.9	75.9	81.5	84.4	86.0	86.3
7–17	89.6	82.0	82.7	87.3	89.7	90.6	90.5

Table S4. Employment rates (%) by educational attainment, age, and age of the youngest child in 1987–2018, single and partnered fathers

	1987–1990	1991–1994	1995–1998	1999–2003	2004–2008	2009–2013	2014–2018
Single fathers							
Total	88.6	73.3	73.9	79.7	82.6	80.1	79.7
Educational attainment							
Lower secondary	86.2	68.7	66.0	69.8	68.9	62.9	59.7
Upper secondary	88.3	71.7	73.3	79.6	83.3	80.3	79.4
Lower tertiary	93.9	82.0	83.4	89.1	91.6	90.8	89.3
Higher tertiary	94.6	92.5	91.3	92.9	92.8	92.1	92.8
Age, years							
18–29	82.4	55.8	57.3	65.7	70.1	63.6	62.5
30–39	89.3	71.8	72.0	78.1	81.2	78.1	78.1
40–49	88.7	75.7	75.8	81.2	83.8	82.1	81.7
Age of the youngest child, years							
0–2	83.2	57.6	59.7	64.2	69.7	68.8	70.1
3–6	88.4	68.7	69.4	76.0	79.4	76.1	77.3
7–17	88.9	74.8	75.3	81.0	83.7	81.6	80.9
Partnered fathers							
Total	93.7	83.0	86.3	90.8	92.4	91.0	91.4
Educational attainment							
Lower secondary	91.8	78.4	79.5	83.6	84.4	80.9	80.0
Upper secondary	92.9	80.7	84.9	89.7	91.5	89.8	90.1
Lower tertiary	96.8	88.5	91.5	95.0	96.2	94.8	95.1
Higher tertiary	97.6	94.7	95.6	96.9	97.1	96.2	96.2
Age, years							
18–29	89.8	71.9	77.2	84.0	86.6	83.9	84.2
30–39	94.6	84.0	87.5	91.8	93.5	92.2	92.2
40–49	93.9	85.0	87.2	91.2	92.7	91.5	92.2
Age of the youngest child, years							
0–2	93.0	80.5	84.8	89.7	91.6	90.0	90.3
3–6	94.2	83.5	87.0	91.3	92.7	91.4	91.9
7–17	94.0	84.6	87.0	91.2	92.8	91.6	92.1

Table S5. Decomposition of the single parent employment gap into category composition and rate effects, mothers

	1987–1990	1991–1994	1995–1998	1999–2003	2004–2008	2009–2013	2014–2018
**Employment rate, %**							
Single mothers	83.0	67.7	65.0	70.1	73.7	73.6	72.3
Partnered mothers	83.6	74.2	75.0	79.8	82.6	84.9	85.2
**Crude difference** (%-points)	0.6	6.5	10.0	9.7	8.9	11.3	12.9
Decomposition effects							
Total for composition	-1.0	0.2	1.0	0.8	1.0	2.2	3.3
Total for rate	1.5	6.4	9.0	8.9	8.0	9.0	9.6
**Composition effects**							
Educational attainment	0.7	1.5	2.5	2.9	3.4	4.1	4.7
Lower secondary	-3.5	-3.9	-4.9	-5.4	-5.4	-4.8	-3.8
Upper secondary	-0.9	-0.6	-0.9	-1.9	-3.5	-5.6	-7.5
Lower tertiary	4.5	4.7	5.9	6.8	7.4	7.8	7.5
Higher tertiary	0.7	1.3	2.4	3.5	4.9	6.6	8.5
Age, years	0.5	1.1	1.0	0.7	0.5	0.6	0.7
18–29	-3.7	-2.5	-1.9	-1.8	-2.0	-2.4	-2.4
30–39	2.2	0.7	0.1	0.7	2.8	3.8	3.0
40–49	1.9	2.9	2.9	1.8	-0.3	-0.9	0.1
Age of the youngest child, years	-2.2	-2.4	-2.5	-2.8	-3.0	-2.4	-2.1
1–2	6.4	4.4	4.5	4.9	6.0	7.1	7.1
3–6	2.7	1.2	0.7	1.5	1.7	1.2	1.2
7–17	-11.4	-8.0	-7.8	-9.3	-10.7	-10.8	-10.4
**Rate effects**							
Educational attainment	0.5	2.1	3.0	3.0	2.7	3.0	3.2
Lower secondary	0.3	0.8	0.9	0.8	0.7	0.7	0.6
Upper secondary	0.2	1.0	1.5	1.5	1.3	1.6	1.8
Lower tertiary	0.0	0.3	0.5	0.5	0.4	0.5	0.6
Higher tertiary	0.0	0.1	0.1	0.1	0.2	0.2	0.3
Age, years	0.5	2.1	3.0	3.0	2.7	3.0	3.2
18–29	0.2	0.4	0.4	0.4	0.4	0.5	0.5
30–39	0.2	1.0	1.5	1.4	1.1	1.3	1.5
40–49	0.1	0.7	1.1	1.1	1.1	1.1	1.2
Age of the youngest child, years	0.5	2.1	3.0	3.0	2.7	3.0	3.2
1–2	0.1	0.5	0.6	0.5	0.5	0.7	0.7
3–6	0.1	0.6	0.9	0.9	0.8	0.9	1.0
7–17	0.4	1.1	1.5	1.5	1.3	1.4	1.5

Table S6. Decomposition of the single parent employment gap into category composition and rate effects, fathers

	1987–1990	1991–1994	1995–1998	1999–2003	2004–2008	2009–2013	2014–2018
**Employment rate, %**							
Single fathers	88.6	73.3	73.9	79.7	82.6	80.1	79.7
Partnered fathers	93.7	83.0	86.3	90.8	92.4	91.0	91.4
**Crude difference (%-points)**	5.2	9.7	12.4	11.0	9.8	10.8	11.7
Decomposition effects							
Total for composition	-0.1	-1.5	-1.3	-1.2	-0.6	-0.1	0.5
Total for rate	5.3	11.2	13.7	12.2	10.4	11.0	11.2
**Composition effects**							
Educational attainment	0.6	1.4	1.6	1.5	1.6	1.9	2.0
Lower secondary	-6.4	-4.8	-5.0	-5.3	-5.2	-5.1	-4.3
Upper secondary	-1.7	-2.0	-2.1	-2.7	-3.6	-3.7	-4.7
Lower tertiary	5.8	5.0	5.1	5.5	5.4	5.1	4.6
Higher tertiary	2.9	3.3	3.5	4.1	5.0	5.7	6.3
Age, years	0.1	-0.5	-0.7	-0.5	-0.3	-0.4	-0.3
18–29	1.2	1.4	1.9	2.1	2.1	1.5	1.2
30–39	1.9	1.4	2.5	4.5	6.3	5.8	4.1
40–49	-3.0	-3.3	-5.0	-7.2	-8.7	-7.7	-5.6
Age of the youngest child, years	-0.7	-2.3	-2.2	-2.1	-1.9	-1.7	-1.2
0–2	20.6	17.3	17.8	19.0	21.1	22.5	22.1
3–6	7.6	5.9	6.8	7.8	6.8	4.8	5.5
7–17	-29.0	-25.6	-26.8	-29.0	-29.8	-28.9	-28.7
**Rate effects**							
Educational attainment	1.8	3.7	4.6	4.1	3.5	3.7	3.7
Lower secondary	0.8	1.3	1.4	1.2	1.1	1.1	0.9
Upper secondary	0.7	1.8	2.2	1.9	1.6	1.9	2.1
Lower tertiary	0.2	0.6	0.7	0.6	0.5	0.4	0.5
Higher tertiary	0.1	0.1	0.2	0.3	0.3	0.3	0.2
Age, years	1.8	3.7	4.6	4.1	3.5	3.7	3.7
18–29	0.3	0.4	0.4	0.4	0.3	0.4	0.4
30–39	0.8	1.8	2.1	1.8	1.3	1.5	1.5
40–49	0.7	1.6	2.1	1.9	1.8	1.8	1.8
Age of the youngest child, years	1.8	3.7	4.6	4.1	3.5	3.7	3.7
0–2	0.5	1.1	1.3	1.3	1.1	1.1	1.0
3–6	0.3	0.8	1.0	0.9	0.7	0.9	0.9
7–17	1.0	1.8	2.2	1.9	1.7	1.7	1.9

Figure S1. Employment rates (%) of single and partnered parents, by age, 1987–2018.

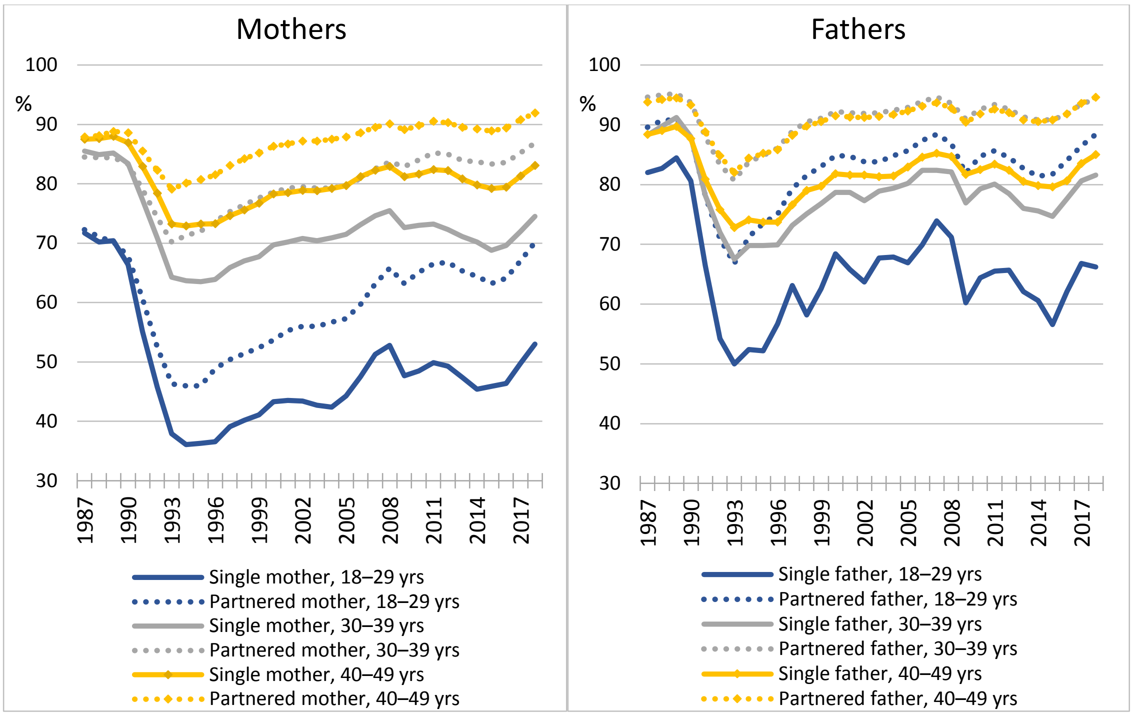

Figure S2. Employment rates (%) of single and partnered parents, by age of the youngest child, 1987–2018.

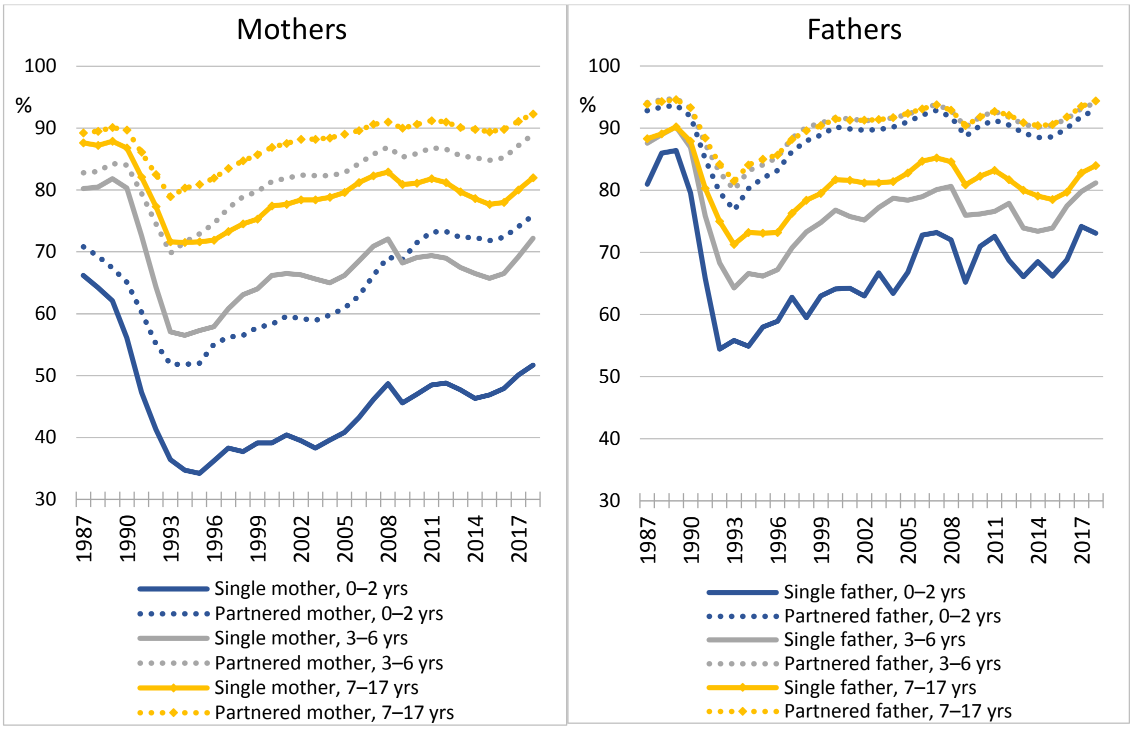

Supplement: Supplementary file 1 — Supplementary file1 (PDF 273 KB) [file 10680_2023_9651_MOESM1_ESM.pdf]
